# Determination of Diphtheria Toxin in Bacterial Cultures by Enzyme Immunoassay

**DOI:** 10.3390/diagnostics12092204

**Published:** 2022-09-11

**Authors:** Maria A. Simonova, Vyacheslav G. Melnikov, Olga E. Lakhtina, Ravilya L. Komaleva, Anja Berger, Andreas Sing, Sergey K. Zavriev

**Affiliations:** 1 Shemyakin-Ovchinnikov Institute of Bioorganic Chemistry of Russian Academy of Sciences, Miklukho-Maklaya 16/10, 117997 Moscow, Russia; 2 National Conciliary Laboratory on Diphtheria, Veterinaerstrasse 2, 85764 Oberschleissheim, Germany; 3 Department of Public Health Microbiology, Bavarian Health and Food Safety Authority, Veterinaerstrasse 2, 85764 Oberschleissheim, Germany

**Keywords:** *Corynebacterium diphtheriae*, *Corynebacterium ulcerans*, diphtheria toxin, ELISA, monoclonal antibodies, level of toxin production

## Abstract

Since diphtheria toxin (DT) is the main virulence factor of *Corynebacterium diphtheriae* and *C. ulcerans*, the detection of DT in corynebacterial cultures is of utmost importance in the laboratory diagnosis of diphtheria. The need to measure the level of DT production (LTP) arises when studying the virulence of a strain for the purpose of diphtheria agent monitoring. To determine the LTP of diphtheria agents, an immunoassay based on monoclonal antibodies (mAbs) has been developed. A pair of mAbs specific to the fragment B of DT was selected, which makes it possible to detect DT in a sandwich ELISA with a detection limit of DT less than 1 ng/mL. Sandwich ELISA was used to analyze 218 liquid culture supernatants of high-, low- and non-toxigenic strains of various corynebacteria. It was shown that the results of ELISA are in good agreement with the results of PCR and the Elek test for the *tox* gene and DT detection, respectively. The diagnostic sensitivity of the assay was approximately 99%, and specificity was 100%. It has been found that strains of *C. ulcerans*, on average, produce 10 times less DT than *C. diphtheriae.* The mAbs used in the ELISA proved to be quite discriminatory and could be further used for the design of the LFIA, a method that can reduce the labor and cost of laboratory diagnosis of diphtheria.

## 1. Introduction

It is easy to get to the bottom of diphtheria—the disease is all about the diphtheria toxin (DT). DT is a potent exotoxin of *Corynebacterium diphtheriae* and *C. ulcerans*, which kills susceptible cells by inhibiting protein synthesis. Specifically, DT transfers the ADP-ribose moiety of NAD to elongation factor EF-2, inactivating it. The ADP-ribosylation activity of DT is determined by the A fragment, and B fragment is required for eukaryotic cell receptor-binding. The toxin repressor (DtxR), a chromosomal regulatory protein, inhibits DT production and derepresses it when Fe^2+^ corepressor is depleted. The phage-encoded DT is the main virulence-associated factor in the disease, responsible for causing diphtheria symptoms, i.e., fever, headache, general malaise, acute tonsillitis with a pseudomembrane over the tonsils, nasopharynx, or even larynx, inflammation and swelling of the cervical lymph nodes (“bull neck”), and systemic complications, including toxin-derived damage to the myocardium, nervous system, and kidneys. Specific prevention of the disease is the vaccination of children with diphtheria toxoid, and the main route of therapy is the administration of hyperimmune equine antitoxic serum (antitoxin). Only toxigenic strains of *C. diphtheriae* and *C. ulcerans* can cause classical respiratory diphtheria, while non-toxigenic strains are not able to do so [1]. Therefore, the accelerated indication of toxin-forming corynebacteria in clinical material is of utmost importance in diphtheria laboratory-based diagnosis, both for managing the individual patient as well as for public health measures.

Laboratory diagnostic tests for toxigenicity of *C. diphtheriae*/*C. ulcerans* are based on qualitative immunological methods [1]. We set ourselves the goal of developing a sensitive and specific test to determine not only the presence, but also the amount of DT produced by the corynebacterial culture. The need to measure the concentration of DT arises when, for instance, studying a relationship between the level of toxin production (LTP) of strains of diphtheria agent, which until recently included only *C. diphtheriae*, and now also includes *C. ulcerans*, and the virulence of the strain. Thus, we found that the *C. diphtheriae* var. *gravis*, ribotype ‘Sankt-Peterburg/Rossija’, MLST type ST8, the diphtheria epidemic clone in Russia and other countries of the former USSR in the 1990s, caused more severe forms of infection in unvaccinated children, compared with the 1980s, when the *C. diphtheriae* var. *mitis*, ribotype ‘Otchakov’, MLST type ST5 was common, and also had a higher LTP. LTP studies were performed using an indirect hemagglutination test with a diagnosticum on sheep erythrocytes sensitized with diphtheria antitoxin, and the result was estimated as the maximum dilution of the liquid bacterial culture, in which hemagglutination still occurred [2]. The high level of DT production in the *C. diphtheriae* ST8 strains was explained by the fact that this epidemic clone had the GCC-> GTC (A147V) mutation in the dtxR gene, that, as shown by chemical mutagenesis studies, modified the regulatory functions of the DtxR protein [3], which, in turn, could lead to an increase in DT expression. However, another research group using the same LTP detection method found that the population of the *C. diphtheriae* ST8 clone was heterogeneous, containing both strongly and weakly toxigenic strains [4]. As can be seen from this example, studies devoted to the development of a reliable method for the determination of LTPs are of importance in assessing the pathogenic potential of corynebacteria.

ELISA remains a very robust and reliable method for the detection of various protein analytes. Previously, enzyme-linked immunosorbent and immunochromatographic methods for detecting DT were developed, the sensitivity of which varied from 0.1 to 4 ng/mL [5,6,7]. These tests were based on a sandwich immunoassay with equine polyclonal antibodies as binding antibodies, and mouse monoclonal antibodies (mAbs) as detecting antibodies. For the determination of LTP, we developed a sensitive immunoassay based only on mAbs, since they have constant properties and, therefore, such a test is easier to standardize.

In addition, we set out to determine whether the mAbs used in ELISA are discriminatory enough to be used in the Lateral Flow Immunoassay (LFIA), a method that can reduce labour and cost of laboratory diagnosis of diphtheria.

## 2. Materials and Methods

### 2.1. Bacterial Strains

A total of 218 strains of corynebacteria for this study (listed in Table S1 in Supplementary Materials) were obtained from the German Conciliary Laboratory on Diphtheria (GCLoD) culture collection. They were both of human and animal origin and isolated in Germany in 2011–2022. The presence of the toxin gene and the DT production were detected by RT-PCR and the Elek test, respectively [8]. The bacterial strains were grown on Columbia Blood Agar (Oxoid, Basingstoke, UK) for 24 h prior to testing.

### 2.2. Sample Preparation

ELISA testing was performed between February 2021 and January 2022. For ELISA testing bacterial strains were cultured on Elek broth [6,7] for 6 h at 37 °C, after which the bacterial cells were removed by filtration through a 0.22-µm-pore-size membrane (Merck Millipore, Burlington, MA, USA). The culture supernatants were stored at −20 °C prior to analysis in the ELISA.

### 2.3. mAb Production and Purification

Cells of mAb-producing hybridomas were used to generate mAbs [9]. Briefly, the producing cells were introduced into BALB/C mice, and preparative amounts of the antibodies were isolated from the ascitic fluids of these mice. The mAbs from the ascitic fluids were purified by affinity chromatography on protein-A-Sepharose (GE Healthcare, Chicago, IL, USA). The ascitic fluid was diluted by four times with the starting buffer (1.5 M glycine and 3 M NaCl, pH 8.9) and applied onto a column that was filled with the affinity sorbent and equilibrated with the same buffer. The mAbs were eluted with a 0.1 M citrate buffer with pH 4.0. The mAb-containing fractions were dialyzed against phosphate buffered saline (PBS). The purity of the mAbs was defined by SDS-PAGE. The mAbs antigen-binding activity was confirmed by indirect ELISA as described previously [9].

### 2.4. Sandwich ELISA

Purified mAbs were biotinylated using the EZ-Link Sulfo-NHS-LC-Biotin reagent (ThermoFisher, Waltham, MA, USA) according to the manufacturer’s instructions. For ELISA, binding antibodies (10 μg/mL in PBS) were adsorbed overnight at 4 °C in the wells (100 μL per well) of a 96-well polystyrene high-binding plate (Costar-Corning, NY, USA). Next, the plate washed 3 times with PBS with 0.05% tween-20 (PBST). Solutions of DT (Sigma-Aldrich, St. Louis, MO, USA) were prepared in PBST with 1% BSA (PBST-BSA) or in Elek broth at various concentrations and added by 100 µL to the wells of the plate with adsorbed antibodies. PBST-BSA or Elek broth without the addition of toxin were used as negative controls. The plate was incubated for 1 h at room temperature on a shaker. Then, the plate was washed 3 times with PBST, 100 µL of a solution of detecting biotinylated mAbs (1 µg/mL in PBST-BSA) was added to each well and incubated for 1 h at room temperature on a shaker. After washing the plate 3 times with PBST, a solution of horseradish peroxidase-labeled streptavidin (BD Biosciences, Franklin Lakes, NJ, USA) in PBST-BSA at a working dilution of 100 μL per well was added and incubated 1 h at room temperature on a shaker. At the end of the incubation, the plate was washed as described above, and 100 µL of the peroxidase substrate, ortho-phenylenediamine (Sigma-Aldrich, St. Louis, MO, USA), was added to each well at a concentration of 1 mg/mL in 1% citrate buffer, pH 4.5, containing 0.05% hydrogen peroxide. The reaction was stopped by adding 50 μL of 2 M sulfuric acid to each well and the color intensity recorded spectrophotometrically (>Packard SpectraCount BS10000, PerkinElmer, Waltham, MA, USA) by determining the optical absorbance at 490 nm.

For detection of DT production in cultures of corynebacteria, culture supernatants were added to the wells of the plate with adsorbed binding antibodies, 100 μL per well. To quantify DT in culture supernatants, samples were diluted 2–100 times with PBST-BSA. The analysis was then carried out as described above. Each sample of the culture supernatant was analyzed in at least duplicates.

### 2.5. Statistical Analysis

Statistical data processing was carried out using the R software environment [10] and specialized packages. The *drc* extension package was used to construct calibration curves and determine DT concentration in culture supernatants [11]. The limit of detection of DT was calculated using the calibration curves and was defined as the concentration of DT corresponding to an optical absorbance value two times higher than the average optical absorbance value of the repeated (at least 10 times) negative control. Differences between samples were tested using the Mann–Whitney U-test and considered statistically significant at *p* < 0.05. ROC analysis by the pROC and ROCR packages was used for the assessment of the accuracy of the method for detecting the toxin, as well as the determination of the threshold of sensitivity of the toxin in bacterial cultures [12,13]. Accuracy, sensitivity, and specificity of qualitative test at each threshold was defined by the formulas: (TP+TN)/(P+N), TP/P, and TN/N, respectively, where P—positive samples, N—negative samples, TP—true positives, TN—true negatives [12].

## 3. Results

### 3.1. Selection of Diagnostic Pair of mAbs

Purified anti-DT mAbs for this study were obtained using hybridoma-producing mAb cells. The purity of mAbs preparations was at least 95% according to the data of SDS-PAGE electrophoresis, and all the mAbs were active in indirect ELISA (data not shown).

The search of diagnostic pairs of mAbs for detection of DT in the sandwich ELISA was carried out. For this, all mAbs were biotinylated and used as detecting antibodies (labeled “biot”). Purified DT manufactured by Sigma was used as a standard. Only mAbs C2G5 and E6B9 worked as a diagnostic pair in the sandwich ELISA. In the C2G5-E6B9biot configuration, the detection limit for DT was 0.4 ± 0.1 ng/mL, so this pair of diagnostic antibodies was used in further studies. It should be noted that both mAbs included in the diagnostic pair were specific for the receptor-binding (B) fragment of DT (Table 1).

### 3.2. Detection of DT in Elek Broth

**Figure 1:**
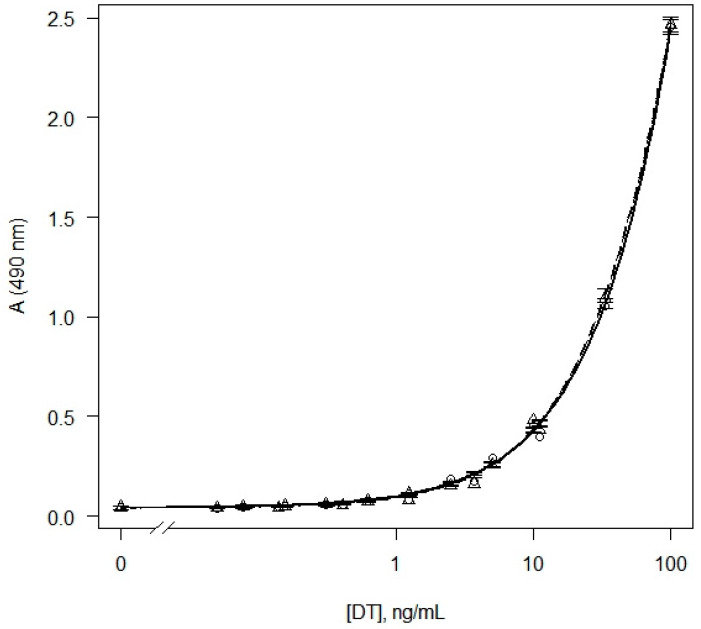
Dose–response curves obtained as results of sandwich ELISA of DT in buffer (circles, solid line) and Elek broth (triangles, dashed line). Model-based standard errors are also shown.

Since the purpose of this work was to determine the DT in liquid bacterial culture, we further studied the effect of the cultivation medium on the results of the analysis of DT. The purified DT was diluted in a standard buffer and Elek broth, and the dose–response curves were analyzed (Figure 1). The detection limit of DT in Elek broth was 0.3 ± 0.1 ng/mL. Background signals in the buffer and Elek medium were not statistically different (*p* = 0.22). Thus, Elek broth did not significantly affect the analysis parameters compared to the buffer.

### 3.3. Sandwich ELISA for Determination of the DT in Bacterial Cultures

Further, we performed the ELISA for detection of the DT in liquid corynebacterial cultures (Table S1 in Supplementary Materials). To determine the background value of the optical absorbance, the average value of the optical absorbance in the samples of the non-inoculated Elek broth was calculated (at least eight replications for each analyzed plate). Next, the ratios of the optical absorbance in the analyzed samples to the background value of the optical absorbance were calculated. Based on the calculated values, as well as the presence of the DT gene and the Elek test results, ROC analysis was performed to determine the predictive ability of the assay, as well as the optimal threshold value of the signal/background ratio. The calculated AUC value was 0.99 (95% confidence interval (CI): 0.97–1.00), which indicates a high predictive ability of the test (Figure S1A in Supplementary Materials). The maximum signal/background ratio, which provides more than 99% accuracy of the qualitative determination of the toxin, was 2.3 (Figure S1B in Supplementary Materials). Thus, the samples for which the signal/background ratio was less than 2.3 were classified as negative, the rest of the samples were classified as positive. For positive samples, a quantitative analysis of DT was performed using calibration curves similar to the curve on Figure 1 (Table S1 in Supplementary Materials).

As can be seen from Table 2, the developed enzyme immunoassay made it possible to detect DT with high accuracy in 218 corynebacterial cultures. At threshold 2.3, the diagnostic sensitivity of the assay was approximately 99%, and specificity was 100%. For all strains, except for one (*C. ulcerans* KL 1902, Table S1 in Supplementary Materials), the results of the ELISA coincided with the results of PCR and the Elek test. All strains producing DT in the Elek test, except for *C. ulcerans* KL 1902 (Table S1 in Supplementary Materials), were ELISA-positive. The KL 1902 strain was *tox*+ and DT-positive, but ELISA-negative. DT-gene-negative *C. diphtheriae*, *C. ulcerans,* and *C. pseudotuberculosis* (diphtheria clade), along with cultures of non-diphtheria corynebacteria, as expected, were found ELISA-negative. Non-toxigenic *tox*-gene positive (NTTB) *C. diphtheriae* and *C. silvaticum* strains were also ELISA-negative.

The average concentrations of DT determined in ELISA-positive *C. ulcerans* cultures (excluding a strain *C. ulcerans* M06-759) were significantly (10 times) lower than the concentrations of DT in cultures of *C. diphtheriae*: 85.0 and 894.0 ng/mL, respectively (*p* < 0.001). *C. ulcerans* M06-759, compared to other strains of *C. ulcerans*, is unique in that it produces an increased amount of DT (2514.8 ng/mL). The reasons for this phenomenon require further study.

## 4. Discussion

The results of this study indicate the high efficiency of the developed ELISA. The only discrepancy was the strain *C. ulcerans* KL 1902, which was producing DT by the Elek test but not by the ELISA. This strain needs to be studied in detail, including by performing whole genome sequencing. It might be speculated that there are some mutations in the toxin gene that disrupt the folding of the toxin molecule and thereby prevent mAbs from binding to the toxin.

Among the 218 strains from GCLoD culture collection used in our study, 21 strains of *C. ulcerans* were *tox* gene positive but initially negative in the Elek test and therefore classified as NTTBs, which are known to circulate worldwide [1,2,4,8,14]. At the same time, all of these 21 strains appeared weakly positive in ELISA. It has been suggested that the current Elek test may not detect the toxin in low-toxigenic strains of *C. ulcerans*. Such observations were first made about 20 years ago [15] by the author of this study (A.S.). The clinical isolate *C. ulcerans* A6361 possessed the DT gene and was Elek test negative. However, the ability of the isolate A6361 to express DT was disclosed using a highly sensitive immunochromatographic strip (ICS) test, which was developed to replace the Elek test [7]. As shown by external quality assessments of the European Diphtheria Surveillance Network (EDSN), most of the laboratories participating in the study also had difficulty testing low-toxigenic strains of *C. ulcerans* with the Elek method [16]. We have made some changes to the immunoprecipitation Elek method (namely, type and concentration of antitoxin, inoculum distance from the antitoxin disk, shape of the bacterial plaques, and position of control strains) and developed a protocol for the optimised Elek test with the capacity to detect all the toxigenic corynebacteria in our study, including those 21 strains with low toxin production [17]. It should be noted that using the previous (less sensitive) modification of the Elek test [8], it was possible to detect every single toxigenic strain of *C. diphtheriae*, even the weakly toxigenic reference strain NCTC 3984. Our study makes this result understandable. The “weakly toxigenic” reference strain of *C. diphtheriae* NCTC 3984 expresses only 4 times less toxin than the control toxigenic strain *C. diphtheriae* NCTC 10648 (878.4 vs. 3370.0 ng/mL), while 21 true weakly toxigenic strains of *C. ulcerans* have average level of toxin production 110 times (!) lower than that of *C. diphtheriae* NCTC 10648 (30.5 vs. 3370.0 ng/mL). It can be concluded that some strains of *C. ulcerans* produce low levels of DT, which were not previously detected by the Elek test and therefore were erroneously classified as non-toxigenic. Hence, ELISA can be used as a reference method to identify isolates with questionable toxin production.

*C. silvaticum* (a recently described species of the diphtheria corynebacteria [18] with a non-expressing DT gene) strains were ELISA-negative. Since the toxigenicity of these GCLoD collection’s *C. silvaticum* cultures was assessed using the previous modification of the Elek test, and that the genetic reason for the non-expression of the DT gene was not revealed even by sequencing, the results of our study proved to be very useful, confirming the lack of ability of *C. silvaticum* to produce a DT. It should be remembered that both ELISA and Elek testing are in vitro methods, therefore, labeling *C. ulcerans* and *C. silvaticum* strains as “poorly toxigenic” or “non-toxigenic” reflects only in vitro data; it cannot be excluded, however, that they are able to produce a significant amount of DT when grown in vivo.

Given that toxigenic *C. ulcerans* were recently recognized as an emerging zoonotic pathogen causing diphtheria-like infections in humans [1,8], the use of sensitive methods for assessing DT production in weakly toxigenic *C. ulcerans* is now of importance. Despite the fact that at present *C. ulcerans* do not cause either severe forms of infection or diphtheria outbreaks, the pathogenetic role of corynebacteria with a low level of toxin production should not be underestimated. The toxin is lethal for susceptible animals as well as unvaccinated humans at doses of 100 ng/kg or less [19].

Moreover, in our recent study 18 allelic variants of the *tox* gene were found across the 291 *tox*+ *C. diphtheriae* isolates. Of these 18 allelic types, 8 contained non-synonymous SNP changes, estimated to be of medium to high structural impact [20]. *C. ulcerans* have a much higher level of *tox* gene variability. They carry about 30 non-synonymous mutations [15] that significantly affect the molecular structure of *C. ulcerans* DT (data not shown). The continually increasing toxin diversity does forecast a real possibility of vaccine escape and antitoxin treatment failure in future. Thus, even if *C. ulcerans* strains are weak toxin producers, mutated strains may become capable of causing disease in people vaccinated against diphtheria.

To conclude, the ELISA is a suitable method for monitoring diphtheria agents by determining LTP. However, using this method in routine practice to detect DT is quite laborious. Thus, there is an urgent need for a simple and reliable test for the rapid indication of toxigenic corynebacteria that can be used in the conventional laboratory. This will significantly reduce the diagnostic time, since it will no longer be necessary to send the clinical specimen or cultures isolated from the patient to the diphtheria reference laboratory. Such a method, for example, can be LFIA based on a pair of mAbs that performed well in this ELISA study.

## Figures and Tables

**Table 1:** Characteristics of monoclonal antibodies to diphtheria toxin (DT).

mAb ID	Isotype	DT Fragment Specificity
C2G5	IgG_1_	B
C12E5	IgG_2b_	A
E4C4	IgG_2a_	A
E6B9	IgG_1_	B
G2D9	IgG_2a_	A
H10B3	n/d	A

n/d—not determined.

**Table 2:** Confusion matrix for the results of qualitative DT detection by sandwich ELISA in corynebacterial cultures.

Total Samples: 218	ELISA: Negative	ELISA: Positive	
Negative samples *:	TN = 114	FP = 0	Total: 114
Positive samples **:	FN = 1	TP = 103	Total: 104
	Total: 115	Total: 103	

FN—false negative; FP—false positive; TN—true negative; TP—true positive. The evaluation was carried out at a threshold signal/background ratio of 2.3. Note: the presence of the toxin gene was detected by RT-PCR; in the absence of the *tox* gene, DT production in most cases was not determined, and strain was considered as negative (*). In the presence of the *tox* gene, DT production was confirmed by the Elek test (**), and if toxin production was not detected in the Elek test, then the strain was considered as negative (*).

## Data Availability

All relevant data are within the manuscript.

## Supplementary Materials

The following supporting information can be downloaded at: https://www.mdpi.com/article/10.3390/diagnostics12092204/s1, Figure S1: ROC-curve (A) and threshold-accuracy dependence curve (B) obtained as results of ROC analysis of ELISA results. AUC, 95% CI for AUC, as well as the signal/background ratio threshold at maximal accuracy are shown. CI—confidence interval. Table S1: The results of the detection of diphtheria toxin by sandwich ELISA in corynebacterial cultures.
