# Supplementary material for: Determination of Diphtheria Toxin in Bacterial Cultures by Enzyme Immunoassay
Source: Diagnostics (Basel). 2022 Sep 11;12(9):2204. doi: 10.3390/diagnostics12092204 (PMC9498204; doi:10.3390/diagnostics12092204)
Supplement: Supplementary file 1 [file diagnostics-12-02204-s001.zip › diagnostics-1890048-supplementary.pdf]

Table S1. The results of the detection of diphtheria toxin (DT) by sandwich ELISA in corynebacterial cultures.

Nr.	GCLoD collection Nr.	Species	*tox* gene (by PCR)	Toxin production (by Elek test)	ELISA results	
					Qualitative	Determined toxin concentration, ng/mL
1	NCTC 3984	*C. diphtheriae*	Pos	Pos	Pos	878.4
2	NCTC 10648	*C. diphtheriae*	Pos	Pos	Pos	3370.0
3	KL 280	*C. diphtheriae*	Pos	Pos	Pos	1865.7
4	KL 339	*C. diphtheriae*	Pos	Pos	Pos	1405.6
5	KL 363	*C. diphtheriae*	Pos	Pos	Pos	858.2
6	KL 364	*C. diphtheriae*	Pos	Pos	Pos	2574.0
7	KL 390	*C. diphtheriae*	Pos	Pos	Pos	1057.1
8	KL 532	*C. diphtheriae*	Pos	Pos	Pos	794.7
9	KL 565	*C. diphtheriae*	Pos	Pos	Pos	762.2
10	KL 603	*C. diphtheriae*	Pos	Pos	Pos	1043.0
11	KL 613	*C. diphtheriae*	Pos	Pos	Pos	1096.3
12	KL 623	*C. diphtheriae*	Pos	Pos	Pos	619.2
13	KL 625	*C. diphtheriae*	Pos	Pos	Pos	581.5
14	KL 631	*C. diphtheriae*	Pos	Pos	Pos	776.4
15	KL 633	*C. diphtheriae*	Pos	Pos	Pos	719.1
16	KL 638	*C. diphtheriae*	Pos	Pos	Pos	299.1
17	KL 652	*C. diphtheriae*	Pos	Pos	Pos	271.6
18	KL 654	*C. diphtheriae*	Pos	Pos	Pos	288.5
19	KL 663	*C. diphtheriae*	Pos	Pos	Pos	221.9
20	KL 670	*C. diphtheriae*	Pos	Pos	Pos	56.4
21	KL 682	*C. diphtheriae*	Pos	Pos	Pos	55.4
22	KL 697	*C. diphtheriae*	Pos	Pos	Pos	402.0
23	KL 839	*C. diphtheriae*	Pos	Pos	Pos	481.8
24	KL 857	*C. diphtheriae*	Pos	Pos	Pos	336.5
25	KL 950	*C. diphtheriae*	Pos	Pos	Pos	417.8
26	KL 956	*C. diphtheriae*	Pos	Pos	Pos	633.9
27	KL 1059	*C. diphtheriae*	Pos	Pos	Pos	1301.4
28	KL 1303	*C. diphtheriae*	Pos	Pos	Pos	843.0
29	KL 1438	*C. diphtheriae*	Pos	Pos	Pos	248.7
30	KL 1648	*C. diphtheriae*	Pos	Pos	Pos	742.3
31	KL 1670	*C. diphtheriae*	Pos	Pos	Pos	769.6
32	KL 1675	*C. diphtheriae*	Pos	Pos	Pos	902.2
33	KL 1686	*C. diphtheriae*	Pos	Pos	Pos	633.9
34	KL 1692	*C. diphtheriae*	Pos	Pos	Pos	788.4
35	KL 1693	*C. diphtheriae*	Pos	Pos	Pos	1066.1
36	KL 1695	*C. diphtheriae*	Pos	Pos	Pos	1150.1
37	KL 1701	*C. diphtheriae*	Pos	Pos	Pos	1251.0
38	KL 1704	*C. diphtheriae*	Pos	Pos	Pos	1325.7
39	KL 1707	*C. diphtheriae*	Pos	Pos	Pos	1040.1

40	KL 1747	*C. diphtheriae*	Pos	Pos	Pos	1139.5
41	KL 1754	*C. diphtheriae*	Pos	Pos	Pos	1189.5
42	KL 1755	*C. diphtheriae*	Pos	Pos	Pos	1255.2
43	KL 1961	*C. diphtheriae*	Pos	Pos	Pos	931.0
44	KL 215	*C. diphtheriae*	Neg	n/d	Neg	0
45	KL 240	*C. diphtheriae*	Neg	n/d	Neg	0
46	KL 286	*C. diphtheriae*	Neg	n/d	Neg	0
47	KL 299	*C. diphtheriae*	Neg	n/d	Neg	0
48	KL 370	*C. diphtheriae*	Neg	n/d	Neg	0
49	KL 356	*C. diphtheriae*	Neg	n/d	Neg	0
50	KL 355	*C. diphtheriae*	Neg	n/d	Neg	0
51	KL 358	*C. diphtheriae*	Neg	n/d	Neg	0
52	KL 377	*C. diphtheriae*	Neg	n/d	Neg	0
53	KL 402	*C. diphtheriae*	Neg	n/d	Neg	0
54	KL 434	*C. diphtheriae*	Neg	n/d	Neg	0
55	KL 461	*C. diphtheriae*	Neg	n/d	Neg	0
56	KL 476	*C. diphtheriae*	Neg	n/d	Neg	0
57	KL 478	*C. diphtheriae*	Neg	n/d	Neg	0
58	KL 479	*C. diphtheriae*	Neg	n/d	Neg	0
59	KL 507	*C. diphtheriae*	Neg	n/d	Neg	0
60	KL 517	*C. diphtheriae*	Neg	n/d	Neg	0
61	KL 557	*C. diphtheriae*	Neg	n/d	Neg	0
62	KL 588	*C. diphtheriae*	Neg	n/d	Neg	0
63	KL 594	*C. diphtheriae*	Neg	n/d	Neg	0
64	KL 596	*C. diphtheriae*	Neg	n/d	Neg	0
65	KL 637	*C. diphtheriae*	Neg	n/d	Neg	0
66	KL 762	*C. diphtheriae*	Neg	n/d	Neg	0
67	KL 765	*C. diphtheriae*	Neg	n/d	Neg	0
68	KL 809	*C. diphtheriae*	Neg	n/d	Neg	0
69	KL 816	*C. diphtheriae*	Neg	n/d	Neg	0
70	KL 828	*C. diphtheriae*	Neg	n/d	Neg	0
71	KL 888	*C. diphtheriae*	Neg	n/d	Neg	0
72	KL 895	*C. diphtheriae*	Neg	n/d	Neg	0
73	KL 920	*C. diphtheriae*	Neg	n/d	Neg	0
74	KL 936	*C. diphtheriae*	Neg	n/d	Neg	0
75	KL 940	*C. diphtheriae*	Neg	n/d	Neg	0
76	KL 948	*C. diphtheriae*	Neg	n/d	Neg	0
77	KL 961	*C. diphtheriae*	Neg	n/d	Neg	0
78	KL 964	*C. diphtheriae*	Neg	n/d	Neg	0
79	KL 965	*C. diphtheriae*	Neg	n/d	Neg	0
80	KL 969	*C. diphtheriae*	Neg	n/d	Neg	0
81	KL 985	*C. diphtheriae*	Neg	n/d	Neg	0
82	KL 1058	*C. diphtheriae*	Neg	n/d	Neg	0
83	KL 1284	*C. diphtheriae*	Neg	n/d	Neg	0
84	KL 1296	*C. diphtheriae*	Neg	n/d	Neg	0
85	KL 1305	*C. diphtheriae*	Neg	n/d	Neg	0

86	KL 1306	*C. diphtheriae*	Neg	n/d	Neg	0
87	KL 1312	*C. diphtheriae*	Neg	n/d	Neg	0
88	KL 1333	*C. diphtheriae*	Neg	n/d	Neg	0
89	KL 1336	*C. diphtheriae*	Neg	n/d	Neg	0
90	KL 1349	*C. diphtheriae*	Neg	n/d	Neg	0
91	KL 1351	*C. diphtheriae*	Neg	n/d	Neg	0
92	KL 1352	*C. diphtheriae*	Neg	n/d	Neg	0
93	KL 1355	*C. diphtheriae*	Neg	n/d	Neg	0
94	KL 1364	*C. diphtheriae*	Neg	n/d	Neg	0
95	KL 1424	*C. diphtheriae*	Neg	n/d	Neg	0
96	KL 1748	*C. diphtheriae*	Neg	n/d	Neg	0
97	KL 1749	*C. diphtheriae*	Neg	n/d	Neg	0
98	KL 1807	*C. diphtheriae*	Neg	n/d	Neg	0
99	KL 1812	*C. diphtheriae*	Neg	n/d	Neg	0
100	KL 1840	*C. diphtheriae*	Neg	n/d	Neg	0
101	KL 1841	*C. diphtheriae*	Neg	n/d	Neg	0
102	KL 179	*C. diphtheriae* (NTTB)	Pos	Neg	Neg	0
103	KL 1810	*C. diphtheriae* (NTTB)	Pos	Neg	Neg	0
104	KL 1854	*C. diphtheriae* (NTTB)	Pos	Neg	Neg	0
105	KL 182	*C. silvaticum* (NTTB)	Pos	Neg	Neg	0
106	KL 1848	*C. silvaticum* (NTTB)	Pos	Neg	Neg	0
107	M06-759	*C. ulcerans*	Pos	Pos	Pos	2514.8
108	KL107	*C. ulcerans*	Pos	Pos	Pos	71.3
109	KL 109	*C. ulcerans*	Pos	Pos	Pos	364.3
110	KL 110	*C. ulcerans*	Pos	Pos	Pos	402.8
111	KL 126	*C. ulcerans*	Pos	Pos	Pos	414.4
112	KL 188	*C. ulcerans*	Pos	Pos	Pos	70.9
113	KL 190	*C. ulcerans*	Pos	Pos	Pos	138.5
114	KL 200	*C. ulcerans*	Pos	Pos	Pos	58.9
115	KL 203	*C. ulcerans*	Pos	Pos	Pos	33.6
116	KL 239	*C. ulcerans*	Pos	Pos	Pos	71.2
117	KL 241	*C. ulcerans*	Pos	Pos	Pos	162.5
118	KL 242	*C. ulcerans*	Pos	Pos	Pos	59.8
119	KL 246	*C. ulcerans*	Pos	Pos	Pos	137.7
120	KL 249	*C. ulcerans*	Pos	Pos	Pos	53.7
121	KL 251	*C. ulcerans*	Pos	Pos	Pos	87.7
122	KL 255	*C. ulcerans*	Pos	Pos	Pos	73.6
123	KL 261	*C. ulcerans*	Pos	Pos	Pos	68.5
124	KL 272	*C. ulcerans*	Pos	Pos	Pos	70.5
125	KL 296	*C. ulcerans*	Pos	Pos	Pos	83.7
126	KL 297	*C. ulcerans*	Pos	Pos	Pos	19.2
127	KL 301	*C. ulcerans*	Pos	Pos	Pos	75.6
128	KL 315	*C. ulcerans*	Pos	Pos	Pos	135.3
129	KL 318	*C. ulcerans*	Pos	Pos	Pos	186.6
130	KL 320	*C. ulcerans*	Pos	Pos	Pos	87.5
131	KL 332	*C. ulcerans*	Pos	Pos	Pos	114.4

132	KL 337	*C. ulcerans*	Pos	Pos	Pos	70.9
133	KL 345	*C. ulcerans*	Pos	Pos	Pos	6.0
134	KL 367	*C. ulcerans*	Pos	Pos	Pos	22.7
135	KL 380	*C. ulcerans*	Pos	Pos	Pos	26.9
136	KL 381	*C. ulcerans*	Pos	Pos	Pos	17.7
137	KL 387	*C. ulcerans*	Pos	Pos	Pos	26.3
138	KL 392	*C. ulcerans*	Pos	Pos	Pos	20.0
139	KL 433	*C. ulcerans*	Pos	Pos	Pos	28.6
140	KL 475	*C. ulcerans*	Pos	Pos	Pos	20.5
141	KL 483	*C. ulcerans*	Pos	Pos	Pos	27.6
142	KL 492	*C. ulcerans*	Pos	Pos	Pos	10.0
143	KL 497	*C. ulcerans*	Pos	Pos	Pos	9.2
144	KL 501	*C. ulcerans*	Pos	Pos	Pos	15.0
145	KL 515	*C. ulcerans*	Pos	Pos	Pos	2.8
146	KL 540	*C. ulcerans*	Pos	Pos	Pos	14.4
147	KL 541	*C. ulcerans*	Pos	Pos	Pos	18.0
148	KL 547	*C. ulcerans*	Pos	Pos	Pos	3.4
149	KL 556	*C. ulcerans*	Pos	Pos	Pos	13.9
150	KL 568	*C. ulcerans*	Pos	Pos	Pos	11.7
151	KL 1294	*C. ulcerans*	Pos	Pos	Pos	44.6
152	KL 1357	*C. ulcerans*	Pos	Pos	Pos	87.0
153	KL 1370	*C. ulcerans*	Pos	Pos	Pos	23.4
154	KL 1750	*C. ulcerans*	Pos	Pos	Pos	167.2
155	KL 1779	*C. ulcerans*	Pos	Pos	Pos	103.0
156	KL 1809	*C. ulcerans*	Pos	Pos	Pos	90.0
157	KL 1819	*C. ulcerans*	Pos	Pos	Pos	182.0
158	KL 1825	*C. ulcerans*	Pos	Pos	Pos	14.4
159	KL 1832	*C. ulcerans*	Pos	Pos	Pos	200.0
160	KL 1853	*C. ulcerans*	Pos	Pos	Pos	181.0
161	KL 1972	*C. ulcerans*	Pos	Pos	Pos	91.0
162	**KL 1902**	***C. ulcerans***	**Pos**	**Pos**	**Neg**	**0**
163	KL 199	*C. ulcerans*	Neg	n/d	Neg	0
164	KL 244	*C. ulcerans*	Neg	n/d	Neg	0
165	KL 274	*C. ulcerans*	Neg	n/d	Neg	0
166	KL 275	*C. ulcerans*	Neg	n/d	Neg	0
167	KL 349	*C. ulcerans*	Neg	n/d	Neg	0
168	KL 366	*C. ulcerans*	Neg	n/d	Neg	0
169	KL 451	*C. ulcerans*	Neg	n/d	Neg	0
170	KL 456a	*C. ulcerans*	Neg	n/d	Neg	0
171	KL 490	*C. ulcerans*	Neg	n/d	Neg	0
172	KL 841	*C. ulcerans*	Neg	n/d	Neg	0
173	KL 853	*C. ulcerans*	Neg	n/d	Neg	0
174	KL 862	*C. ulcerans*	Neg	n/d	Neg	0
175	KL 876	*C. ulcerans*	Neg	n/d	Neg	0
176	KL 941	*C. ulcerans*	Neg	n/d	Neg	0
177	KL 1017	*C. ulcerans*	Neg	n/d	Neg	0

178	KL 1021	*C. ulcerans*	Neg	n/d	Neg	0
179	KL 1295	*C. ulcerans*	Neg	n/d	Neg	0
180	KL 1321	*C. ulcerans*	Neg	n/d	Neg	0
181	KL 1335	*C. ulcerans*	Neg	n/d	Neg	0
182	KL 1340	*C. ulcerans*	Neg	n/d	Neg	0
183	KL 1361	*C. ulcerans*	Neg	n/d	Neg	0
184	KL 1447	*C. ulcerans*	Neg	n/d	Neg	0
185	KL 1455	*C. ulcerans*	Neg	n/d	Neg	0
186	KL 1465	*C. ulcerans*	Neg	n/d	Neg	0
187	KL 1466	*C. ulcerans*	Neg	n/d	Neg	0
188	KL 1474	*C. ulcerans*	Neg	n/d	Neg	0
189	KL 1540	*C. ulcerans*	Neg	n/d	Neg	0
190	KL 1563	*C. ulcerans*	Neg	n/d	Neg	0
191	KL 1574	*C. ulcerans*	Neg	n/d	Neg	0
192	KL 1578	*C. ulcerans*	Neg	n/d	Neg	0
193	KL 1583	*C. ulcerans*	Neg	n/d	Neg	0
194	KL 1596	*C. ulcerans*	Neg	n/d	Neg	0
195	KL 1622	*C. ulcerans*	Neg	n/d	Neg	0
196	KL 1649	*C. ulcerans*	Neg	n/d	Neg	0
197	KL 1664	*C. ulcerans*	Neg	n/d	Neg	0
198	KL 1665	*C. ulcerans*	Neg	n/d	Neg	0
199	KL 1694	*C. ulcerans*	Neg	n/d	Neg	0
200	KL 1743	*C. ulcerans*	Neg	n/d	Neg	0
201	KL 1802	*C. ulcerans*	Neg	n/d	Neg	0
202	KL 1808	*C. ulcerans*	Neg	n/d	Neg	0
203	KL 1971	*C. ulcerans*	Neg	n/d	Neg	0
204	HB 457	*C. pseudotuberculosis*	Neg	Neg	Neg	0
205	DSM 7180	*C. pseudotuberculosis*	Neg	Neg	Neg	0
206	DSM 20689	*C. pseudotuberculosis*	Neg	Neg	Neg	0
207	KL 1634	*C. pseudotuberculosis*	Neg	Neg	Neg	0
208	KL 1635	*C. pseudotuberculosis*	Neg	Neg	Neg	0
209	KL 128	*C. accolens*	Neg	n/d	Neg	0
210	KL 129	*C. accolens*	Neg	n/d	Neg	0
211	KL 1452	*C. pseudodiphtheriticum*	Neg	n/d	Neg	0
212	KL 1522	*C. amycolatum*	Neg	n/d	Neg	0
213	KL 1536	*C. striatum*	Neg	n/d	Neg	0
214	KL1566	*C. amycolatum*	Neg	n/d	Neg	0
215	KL 1680	*C. amycolatum*	Neg	n/d	Neg	0
216	KL 1684	*C. amycolatum*	Neg	n/d	Neg	0
217	KL 1711	*C. amycolatum*	Neg	n/d	Neg	0
218	KL 1715	*C. tuberculostearicum*	Neg	n/d	Neg	0

Neg – negative; Pos – positive; NTTB – non-toxigenic *tox*-negative. Highlighted in bold - strain KL 1902 with "unusual" properties. Note - in the absence of the *tox* gene, DT in most cases was not determined (n/d).

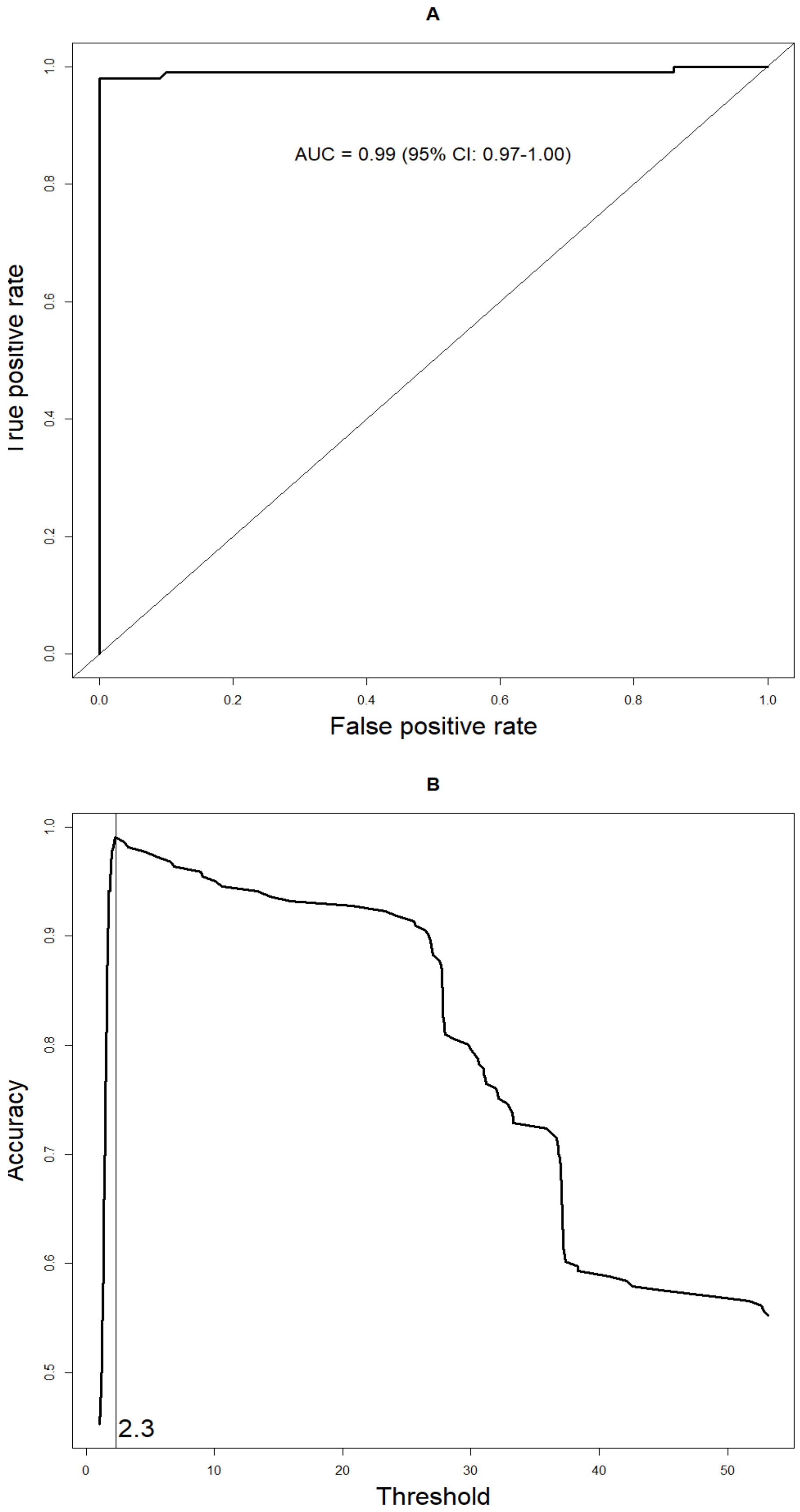

Figure S1. ROC-curve (A) and threshold-accuracy dependence curve (B) obtained as results of ROC analysis of ELISA results. AUC, 95% CI for AUC, as well as the signal/background ratio threshold at maximal accuracy are showed. CI – confidence interval.
